# Modulation of initial movement for double potential targets with specific time constraints

**DOI:** 10.1038/s41598-021-01777-3

**Published:** 2021-11-18

**Authors:** Ryoji Onagawa, Kazutoshi Kudo

**Affiliations:** 1 Laboratory of Sports Sciences, Department of Life Sciences, Graduate School of Arts and Sciences, The University of Tokyo, Tokyo, Japan; 2 Research Fellow of Japan Society for the Promotion of Science, Tokyo, Japan; 3 Faculty of Science and Engineering, Waseda University, Tokyo, Japan

**Keywords:** Decision, Human behaviour

## Abstract

In goal-directed behavior, individuals are often required to plan and execute a movement with multiple competing reach targets simultaneously. The time constraint assigned to the target is an important factor that affect the initial movement planning, but the adjustments made to the starting behavior considering the time constraints specific to each target have not yet been clarified. The current study examined how humans adjusted their motor planning for double potential targets with independent time constraints under a go-before-you-know situation. The results revealed that the initial movements were modulated depending on the time constraints for potential targets. However, under tight time constraints, the performance in the double-target condition was lower than the single-target condition, which was a control condition implemented to estimate performance when one target is ignored. These results indicate that the initial movement for multiple potential targets with independent time constraints can be modified, but the planning is suboptimal.

## Introduction

In goal-directed behaviors, individuals are often required to perform movements under tight time constraints. This is especially pertinent to many sports, where decisions are made under severe time constraints, e.g., a defender responding to an opponent's dribble in ball games such as soccer or basketball. In these situations, motor plans are selected considering multiple possibilities for motor goals; however, the action often needs to be initiated and continued before a single motor goal is defined. Several studies have investigated motor planning for multiple potential goals using the “Go-before-you-know paradigm,” in which a participant is required to launch a movement with multiple potential goals and the final goal is revealed after movement development (i.e., movement onset or reaching a given threshold)^1–10^. In general, when there are multiple potential goals at movement onset, the initial movements were found to be directed toward the average direction of the potential targets^1–3,5,6,11,12^.

The performance of a goal-directed movement is traditionally conceptualized in two discrete phases: the planning phase and the execution phases^13^. Based on this discrete view, several models have been created to solve the redundancy problem (for instance, when reaching for an object, there are numerous hand trajectories, joint movements, and muscle activation patterns that can be executed on a single target). To solve this problem, optimization based on various costs, such as jerks^14^, torque-changes^15^, variabilities of the final hand position^16^, and effort^17,18^ have been proposed, and these perspectives have contributed greatly to our understanding of human motor control principles. However, although these traditional serial models^19^ assume that we first select a goal and then specify and prepare the corresponding goal-directed movement, selection and specification can operate as a continuous and parallel processes^20,21^. For example, the behavioral findings from recent studies have revealed that the movement trajectories in the simultaneous presence of multiple potential targets are directed in directions where no target exists^1^. Furthermore, human and primate neurophysiology studies have reported that competing reach targets induce separate neural representations corresponding to each target in sensory-motor brain areas before one of the targets is selected^22–25^. On the contrary, a recent study reported that premotor cortex in macaque plans only one option at a time when facing with two potential reach targets^26^. How multiple goals are considered and converge into a single action has not been fully clarified so far.

Many behavioral studies using go-before-you-know tasks (i.e., tasks in which participants are presented with a large number of potential reaching targets simultaneously before knowing the final target location and are required to initiate a reaching movement to a competing target) have confirmed that humans initiate reaching movements toward the average of the potential targets^1,10,12^. However, such averaging behavior is less likely to be selected when the advantage of the strategy disappears, such as when the distance between targets is large^7^, when severe constraints on speed are imposed^8^, or when the information on the targets is updated in stages^27^. These findings suggest that the averaging behavior in motor planning in the presence of multiple goals may not exist as a control policy in itself, but is rather a behavior that reflects optimization for task accomplishment.

In a go-before-you-know-paradigm, the effect of time constraints assigned to potential targets on motor planning has not been sufficiently investigated. The time constraint is a critical factor because the motor target is often time-constrained, and the time constraint affects the possible movement dynamics. In addition, the effect of asymmetry between potential targets on motor planning has not yet been discussed. In many situations, different motor targets have specific time constraints, and "how do humans execute movements in situations where targets with different time constraints exist simultaneously?" seems to be an important question. Therefore, in the current study, we investigated how to adjust the initial movements according to the time constraints assigned to each potential target.

The time constraint is an effective experimental control variable for examining how asymmetry in potential targets is reflected in motor planning. Although this approach of experimentally manipulating time constraints is similar to that used by Wong and Haith^8^, who restricted the movement velocity, or that used by Hesse et al.^28^, who manipulated the positioning of the target to manipulate the optimality of the averaging strategy, two reasons exist as to why manipulating time constraints may be effective for further understanding of behaviors. First, unlike constraints on movement speed, time constraints do not directly constrain the initial movement itself. Since the participants themselves are free to choose the initial movement, manipulation of the time constraint would be more appropriate for examining the adaptability of the motor planning to the given target information. Next, specific time constraints can be assigned to each target when manipulating the time constraint, thus creating an asymmetry in the temporal values of potential targets. Such manipulation of the time constraint can be used to examine how humans plan their movements to account for asymmetric temporal values of potential targets, while this approach is difficult to employ in studies on movement velocity.

Although motor planning for potential targets has been suggested to reflect optimization of success probability^8^, the extent of this optimality has not been clarified. The direction and velocity of the initial movement, as well as the corrective actions after the movement, are considered to reflect the strategy selected in advance by the participants. In this case, an important decision about the strategy for two potential targets is whether to focus on one target (i.e., a predetermined strategy) or to take both targets into account (i.e., a choice-reaction strategy). Previous studies confirmed that humans prefer a choice-reaction strategy even in situations with severe spatiotemporal constraints to sufficiently accomplish a task^28,29^. A similar bias may exist in the selection of strategies when encountering asymmetric time constraints in the go-before-you-know task. Thus, the present study examined whether pre-determined or choice-reaction strategies can be optimally selected to maximize the success rate under extremely tight time constraints by comparing the performance when two targets are present with the performance when only one target is present.

Therefore, the current study used a go-before-you-know task with two targets to examine how a combination of the time constraints assigned to each target is considered in motor planning. Participants started the movement with the time constraints of each target known in advance, and the final target was specified after the movement onset. Participants were considered successful when they reached the final target within the time constraint. The time constraint for each target was randomly assigned for each trial in the range of 200–1000 ms. Participants were required to maximize the success probability within the set (50 trials). As a control condition, participants also performed trials with only one target before the start of the movement. The kinematic properties (i.e., direction and velocity) and optimality of the planning of the initial movement were tested by examining the variations in the direction and velocity of the initial movement in the double-target condition depending on the time constraints, and by comparing the initial movement and performance in relation to the number of potential targets. More specifically, we first examined the changes in motor patterns related to combinations of time constraints, based on movement trajectories and the bivariable histograms of and kinematic properties of the initial movement. We also categorized the patterns of initiating actions in a data-driven manner using k-means clustering and examined how the ratio of occurrences of each pattern changes depending on the combination of time constraints. In addition, we examined the variation in the initial movement pattern and performance depending on the time constraint and number of targets.

## Methods

### Participants

Twelve right-handed neurologically healthy participants (age: 22.7 ± 3.1 years, ten men) were recruited. All patients had normal or corrected-to-normal vision, were naive to the objectives of this study, and provided written informed consent. This study was approved by the Ethics Committee of the Graduate School of Arts and Sciences, University of Tokyo. All experimental procedures adhered to approved guidelines for experimental procedures. Informed consent was obtained from each participant before the experiments in a written format.

### Experimental setup

The participants sat in a quiet, dim room. A pen tablet with sufficient workspace to measure the subjects’ arm reach movement (Wacom, Intuos 4 Extra Large; workspace: 488 × 305 mm) was set on the table. A monitor (I-O DATA, KH2500V-ZX2; 24.5 inches, 1920 × 1080 pixels, vertical refresh rate, 240 Hz) was set for stimulus presentation with an approximately 30° gradient angle over the pen-tablet. The participants manipulated a cursor on a screen whose position was transformed from the position of the pen. The time elapsed from the movement onset and the location of the cursor on the monitor were sampled at 240 Hz. All stimuli were controlled using the Psychophysics Toolbox of MATLAB (MathWorks, Natick, MA, USA).

### Experimental task

The participants performed a task-modified version of a go-before-you-know paradigm, in which two potential targets were presented on the screen and one of the targets was revealed as the final target only after the participant launched his/her movements. These targets appeared 20 cm away from the start position and were presented on either side of the midline at + 30° and − 30°. Independent time constraints were randomly assigned to each target in each trial. The time constraints were uniformly distributed over a range of 200 to 1000 ms. For the first six participants, we used a combination of uniform distributions to set the time constraint, which resulted in a slight (but not major) bias in number of trials corresponding to combination of time constraints. For the remaining six participants, to eliminate bias, we used a pseudo-random number with an equal number of trials in a 5 × 5 bin. The direction of the true target (+ 30° or − 30°) was equally randomized in each condition and set. Each set included 50 trials.

**Figure 1:**
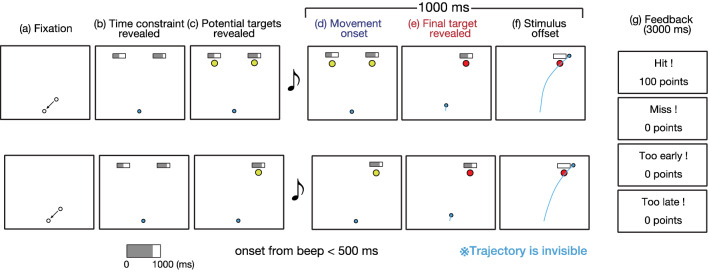
Sequence of the experimental task. The top row shows the double-target condition. The bottom row shows the single-target condition. (**a**) First, participants move the cursor to the start position. (**b**) After the presentation of the time constraint indicator, (**c**) the potential targets are presented. (**d**) 450–1000 ms later, participants were required to start the movement after the sound stimulus. After the movement onset, the gray area of the indicator reduces linearly with time. (**e**) When the participant's movement onset is detected, the final target is presented. (**f**) The stimulus disappeared 1000 ms after the movement onset. Participants acquired 100 points if they met the movement onset criteria and passed the final target within the time constraint assigned to the final target. (**g**) Feedback on successes, failures, and scores was provided after each movement.

Figure 1 shows the sequence of the trial. The sequence of tasks was almost the same for the double-target and single-target conditions. To begin the trial, participants moved a cursor (white frame circle, radius: 0.5 cm) to a start position (white frame circle, radius: 0.5 cm) presented on a screen (Fig. 1a). When the cursor reached the start position, it turned into a blue circle. Subsequently, the time bar indicating the time constraints assigned for each target was presented above the potential target position (Fig. 1b). After 1000 ms, the potential targets are presented in Fig. 1c. After a random interval (450–1000 ms), an auditory beep cued the participant to initiate a movement. Participants were required to initiate a movement up to 500 ms after the beep cue. When the cursor was 1 cm away from the start position, the true target changed to a filled yellow circle, and the other target disappeared (Fig. 1d,e). After movement onset, the area of the gray bar decreased over time. When the cursor moved through the true target within a given time constraint for the target, the participants acquired 100 points. After the stimuli disappeared (1000 ms after movement onset, Fig. 1f), the results of the movements (“Hit” or “Miss”) and the scores (0 points for a missed trial or 100 points for a successful trial) were presented as feedback (Fig. 1g). If movement onset time was more than 500 ms or less than 0 ms (i.e., movement onset was faster than a beep cue), “Too late” or “Too early” was presented on the screen as feedback, respectively. In this case, the participants acquired no points. The participants were instructed to maximize the average score for each set.

First, the double-target condition was performed for six sets of 50 trials. Then, the single-target condition was performed for two sets of 50 trials. Sufficient rest was taken between the sets to avoid fatigue.

### Data analysis

The observed data were analyzed using programs written in MATLAB software (MathWorks, Natick, MA, USA). The data obtained in the last four sets in the double-target trials (200 trials) and all sets in the single-target trials (100 trials) were used for analysis. The reason for excluding the first two sets in the double-target condition was to examine the behavior when they were sufficiently familiar with the task. The cursor positions (horizontal position:$Xc\left(t\right)$, vertical position: $Yc\left(t\right)$) at each time point ($t$) were calibrated using a second-order, zero-phase-lag, low-pass Butterworth filter with a cutoff frequency of 6 Hz.

The movement onset time was identified using the cursor distance ($d(t)=\sqrt{{x}_{c}{(t)}^{2}+{y}_{c}{(t)}^{2}}$) 5% of the target distance from the start position (0.5 cm). Reach time was defined as the time when the cursor distance $d(t)$ was longer than the distance from the start position to the target (i.e., 20 cm). The cursor angle at each time point (t) was determined as the angle between the vector from the start position to the cursor position and the horizontal vector. The initial movement direction (IMD) was determined as the cursor angle at 100 ms after the movement onset, while the initial movement velocity (IMV) was determined as the movement velocity 100 ms after movement onset.

### Clustering of the initial movement

To classify the characteristics of the initial movement, k-means clustering was used to classify the initial movement into three clusters based on the combination of the IMD and IMV. The advantage of using machine-learning clustering without determining the angle threshold was that it was flexible enough to deal with the directional dependency of arm reaching.

### Comparison of initial movement and performance between the double-target and single-target conditions

The combinations of time constraints for the left and right targets were classified as 5 × 5, and the means of IMV, IMD, and |⊿IMD| were calculated for each. |⊿IMD| is defined as the absolute value of the angle difference between the IMD and vertical vector. In addition, within the same classification of time constraints, the means of temporal performance, arrival direction accuracy, and overall performance were calculated for each individual. Temporal performance denotes the probability that the reach time is less than the time constraint, regardless of whether the target was hit or not. The arrival direction accuracy indicates the probability that the arrival direction matches the correct target and does not consider whether the target can be hit correctly. Overall performance indicates the probability of success (i.e., whether the hit is accurate, in time). These indices were compared between double-target and single-target conditions.

### Statistical analysis

To test the modulation of the initial movement depending on the time constraints, three-way repeated-measures ANOVAs (3 [cluster] × 5 [left time constraint] × 5 [right time constraint]) of the occurrence probability and two-way repeated-measures ANOVAs (5 [time constraints on left target] × 5 [time constraints on right target] of the IMV, IMD, and |⊿IMV|, and post-hoc tests were conducted. In addition, under the condition where one of the time constraints was the most severe, the effect of the other time constraint condition and the number of targets, two-way repeated-measures ANOVAs (2[number of targets] × 5[time constraints]) of the overall performance were conducted with either the left or right time-constraint fixed.

## Results

### Modulation of the initial movement according to the combination of time constraints

**Figure 2:**
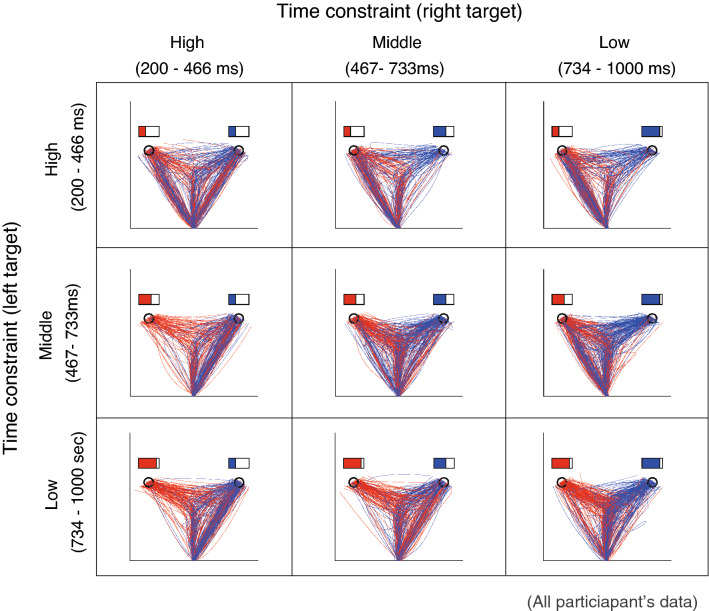
Movement trajectories according to combinations of the time constraints. The movement trajectories according to the time constraint of all participants in the double-target condition are shown. The color is based on the final target (red: final target is left, blue: final target is right). This figure confirmed that the trajectories of the participants' movements are modulated according to the combination of time constraints. For combinations of equivalent time constraints, the trajectories seemed to be bilaterally symmetrical. On the other hand, in situations with different time constraints, the frequency of the initial movement to the target with shorter time constraints seemed to be higher.

First, we descriptively examined the changes in the initial movement according to the combination of time constraints. Figure 2 shows the trajectories of cursors classified by time constraints, including the data of all participants (the trajectories of each participant are shown in the Supplementary information). This figure suggests that the selected trajectory modulates according to the time constraint. From a qualitative perspective, the initial movement was divided into three main directions: center direction, left target direction, and right target direction. While movement in the center direction was observed within any time constraint, movement in the target directions was mainly observed in the direction with tighter time constraints.

**Figure 3:**
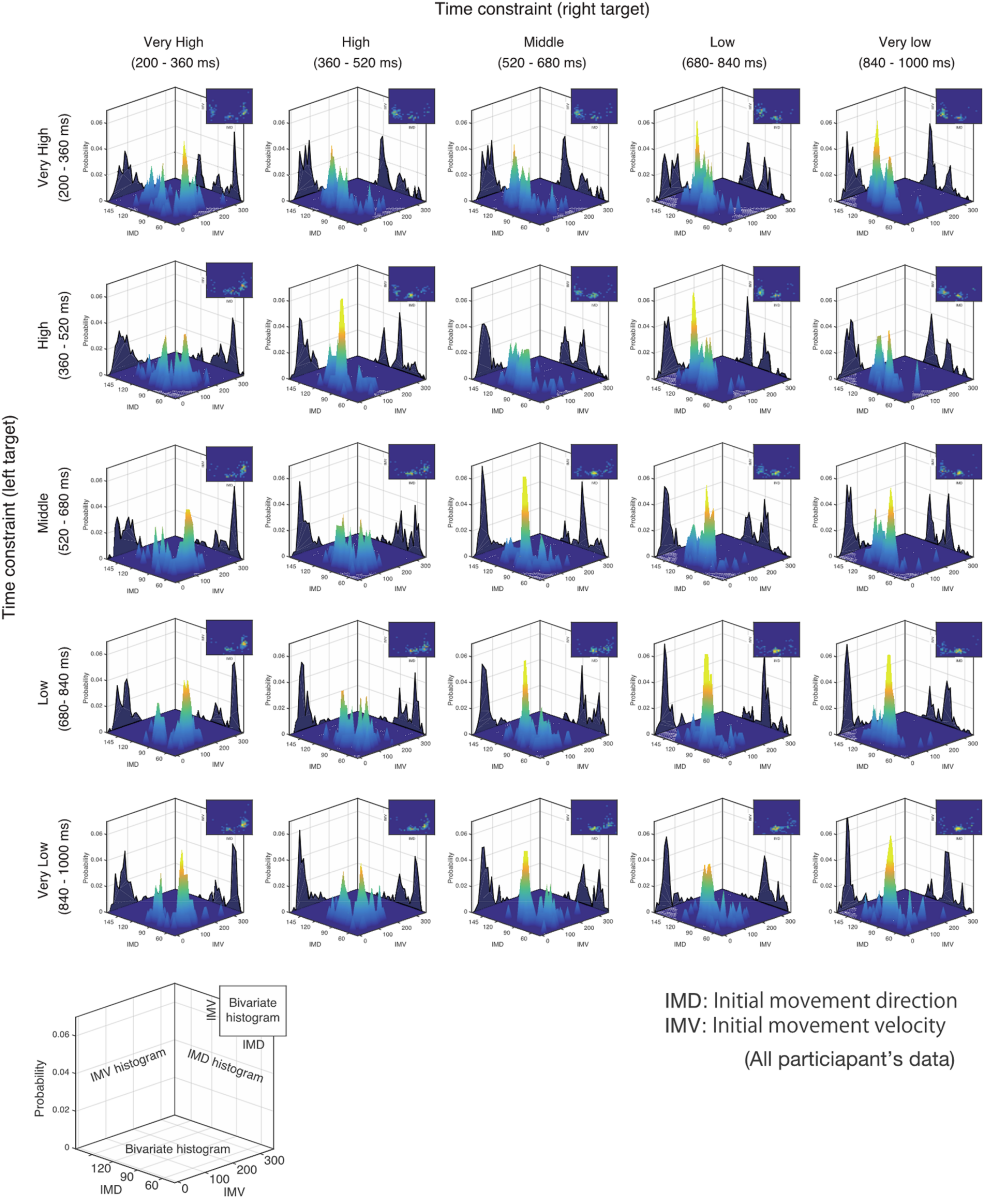
Bivariate histograms of the initial movement direction (IMD) and the initial movement velocity (IMV) according to the combinations of the time constraints. The bivariate histograms of the IMD and the IMV according to the time constraint in the double-target condition are shown. The center of each box shows the bivariate histogram, and the upper right corner shows the same histogram viewed from the vertical direction. As in Fig. 2, this Figure shows the change in the direction and velocity of the initial movement depending on the time constraint. In particular, the diagonal line from the upper left to the lower right shows a high frequency of initial movement in the center direction and a symmetrical distribution along 90° of the IMD. On the other hand, in the conditions where the difference in time constraints is large (lower left or upper right panel), the frequency of the initial movement in the target direction with tighter time constraints is high, although initial movement in the center direction also exists.

Modulation dependent on the time constraint was also clearly seen in the bivariate histograms of the initial movement direction (IMD) and initial movement velocity (IMV), as shown in Fig. 3. More specifically, when the time constraints were comparable, a high frequency of initial movement in the center direction and a symmetrical distribution along 90° of the IMD were confirmed. On the other hand, under conditions where the difference in time constraints was large (lower left or upper right panel), the frequency of the initial movement in the target direction with tighter time constraints was high, although initial movement in the center direction also existed.

**Figure 4:**
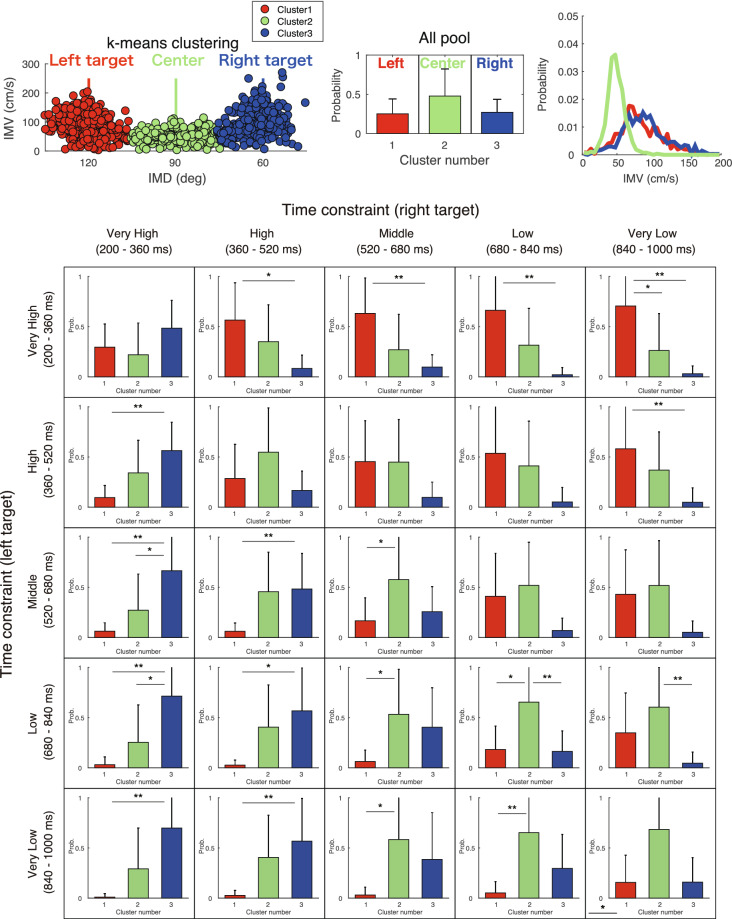
Clustering of the initial movement and the probability of appearance of movement patterns according to time constraints. The upper left panel shows a scatter plot of the movement direction and velocity of the initial movement, including data from all participants. The data were classified into three clusters (red, green, and blue) by k-means clustering for the bivariate initial movement variables (i.e., the IMD and IMV). The upper center panel shows the between-participant mean of the probability of occurrence of each cluster under all time constraints. Error bars are between-participant standard deviations. The upper right panel shows the histograms of IMV in each cluster. It was confirmed that the IMV in the center direction (green) was slower than that in the target directions (red and blue). The bottom panels show the between-participant average of the probability of occurrence of each cluster, depending on the time constraint. This figure shows that when the time constraints are equal, the occurrence probability of the intermediate direction (green) is higher, and with differences in the time constraints, the occurrence probability of the initial movement to the target direction (red and green) with a shorter time constraint is higher.

To quantitatively demonstrate the qualitative observations highlighted in Fig. 3, the initial movement patterns (IMD and IMV) were classified into three clusters by using k-means clustering, and the percentage of occurrence of each pattern was compared.  This analysis could more clearly quantify the modulation of initial movement patterns. This analysis addresses the problem that the average values of IMD were equivalent when the movement was directed to either the left or right target with the same frequency, or when the movement was directed at the center. Figure 4 shows the scatter plots of the IMD and IMV, including all data for all participants, and the occurrence probability of each cluster for each combination of time constraints. This result quantitatively illustrates the qualitative observations shown in Fig. 3. The scatter plots (top left panel) and the histograms of IMV in each cluster (top right panel) indicated that the initial movement in the center direction was relatively slow, and the initial movement in the target direction was relatively fast. This difference in velocity may reflect differences in the subsequent correction strategy.

A three-way repeated-measures ANOVA (3 [cluster] × 5 [left time constraint] × 5 [right time constraint]) on the occurrence probability revealed a three-way interaction (*F*_[8,88]_  = 2.906, η^2^_p_ = 0.209, *p* < 0.001). Post-hoc one-way ANOVAs (3 [cluster]) and multiple comparisons in each combination of time constraints are shown in Tables 1 and 2, which reveals that the occurrence probability of each cluster depending on time constraints suggested that the proportion of movement directions (i.e., left, center, or right) varied depending on the combination of time constraints assigned to each target. In the condition where the time constraint did not differ between potential targets, the middle direction was selected more frequently, and when the time constraint differed, the target direction with a shorter time constraint was selected more frequently. These results showed that participants selected different initial movements depending on the time constraint.

**Table 1:** One-way repeated-measures ANOVA (3 [cluster]) on the occurrence probability.

Time constraint	*F* [2,22]	*p*	partial η^2^
Very high (right), Very high (left)	0.224	0.166	0.151
High (right), Very high (left)*	4.801	0.019*	0.304
Middle (right), Very high (left)**	6.786	0.005**	0.382
Low (right), Very high (right)**	8.788	0.002**	0.444
Very low (right), Very high (left)**	9.181	0.001**	0.455
Very High (right), High (left)**	6.583	0.006**	0.374
High (right), High (left)	2.643	0.094	0.194
Middle (right), High (left)	2.715	0.088	0.198
Low (right), High (left)*	3.475	0.049*	0.24
Very low (right), High (left)*	5.033	0.016*	0.314
Very high (right), Middle (left)**	8.662	0.002**	0.441
High (right), Middle (left)*	4.665	0.02*	0.298
Middle (right), Middle (left)*	3.746	0.04*	0.254
Low (right), Middle (left)	3.447	0.05	0.239
Very low (right), Middle (left)*	3.596	0.045*	0.246
Very High (right), Low (left)**	10.044	< .001**	0.477
High (right), Low (left)*	5.102	0.015*	0.317
Middle (right), Low (left)*	3.874	0.036*	0.26
Low (right), Low (left)**	7.306	0.004**	0.399
Very low (right), Low (left)*	5.79	0.01*	0.345
Very high (right), Very low (left)**	8.324	0.002**	0.431
High (right), Very low (left)*	5.501	0.012*	0.333
Middle (right), Very low (left)*	4.212	0.028*	0.277
Low (right), Very low (left)**	7.73	0.003**	0.413
Very low (right), Very low (left)**	7.003	0.004**	0.389

*< 0.05, **< 0.01.

**Table 2:** Multiple comparisons (3 [cluster]) on the occurrence probability.

Multiple comparison			
Time constraints	Compared clusters	*t* [11]	*p*_bonf_
Very high (right), Very high (left)	1 vs 2	0.547	1
	1 vs 3	− 1.371	0.553
	2 vs 3	− 1.918	0.205
High (right), Very high (left)	1 vs 2	1.379	0.545
	1 vs 3*	3.093	0.016*
	2 vs 3	1.713	0.302
Middle (right), Very high (left)	1 vs 2	2.447	0.069
	1 vs 3**	3.609	0.005**
	2 vs 3	1.162	0.773
Low (right), Very high (left)	1 vs 2	2.264	0.101
	1 vs 3**	4.188	0.001**
	2 vs 3	1.924	0.202
Very low (right), Very high (left)	1 vs 2*	2.772	0.033*
	1 vs 3**	4.216	0.001**
	2 vs 3	1.444	0.488
Very high (right), high (left)	1 vs 2	− 1.906	0.21
	1 vs 3**	− 3.627	0.004**
	2 vs 3	− 1.721	0.298
High (right), High (left)	1 vs 2	− 1.542	0.412
	1 vs 3	0.706	1
	2 vs 3	2.248	0.105
Middle (right), High (left)	1 vs 2	0.026	1
	1 vs 3	2.031	0.164
	2 vs 3	2.005	0.172
Low (right), High (left)	1 vs 2	0.656	1
	1 vs 3	2.539	0.056
	2 vs 3	1.883	0.219
Very low (right), High (left)	1 vs 2	1.251	0.673
	1 vs 3*	3.15	0.014*
	2 vs 3	1.9	0.212
Very High (right), Middle (left)	1 vs 2	− 1.427	0.503
	1 vs 3**	− 4.1	0.001**
	2 vs 3*	− 2.672	0.042*
High (right), Middle (left)	1 vs 2	− 2.553	0.054
	1 vs 3*	− 2.729	0.037*
	2 vs 3	− 0.175	1
Middle (right), Middle (left)	1 vs 2*	− 2.605	0.049*
	1 vs 3	− 0.574	1
	2 vs 3	2.031	0.164
Low (right), Middle (left)	1 vs 2	− 0.609	1
	1 vs 3	1.907	0.209
	2 vs 3	2.516	0.059
Very low (right), Middle (left)	1 vs 2	− 0.476	1
	1 vs 3	2.048	0.158
	2 vs 3	2.524	0.058
Very high (right), Low (left)	1 vs 2	− 1.434	0.497
	1 vs 3**	− 4.394	< .001**
	2 vs 3*	− 2.961	0.022*
High (right), Low (left)	1 vs 2	− 2.176	0.122
	1 vs 3*	− 3.113	0.015*
	2 vs 3	− 0.937	1
Middle (right), Low (left)	1 vs 2*	− 2.692	0.04*
	1 vs 3	− 1.958	0.189
	2 vs 3	0.734	1
Low (right), Low (left)	1 vs 2*	− 3.244	0.011*
	1 vs 3	0.129	1
	2 vs 3**	3.373	0.008**
Very low (right), Low (left)	1 vs 2	− 1.56	0.399
	1 vs 3	1.839	0.238
	2 vs 3**	3.399	0.008**
Very high (right), Very low (left)	1 vs 2	− 1.66	0.333
	1 vs 3**	− 4.058	0.002**
	2 vs 3	− 2.398	0.076
High (right), Very low (left)	1 vs 2	− 2.014	0.169
	1 vs 3**	− 3.289	0.01**
	2 vs 3	− 1.275	0.647
Middle (right), Very low (left)	1 vs 2*	− 2.864	0.027*
	1 vs 3	− 1.838	0.239
	2 vs 3	1.027	0.947
Low (right), Very low (left)	1 vs 2**	− 3.909	0.002**
	1 vs 3	− 1.587	0.38
	2 vs 3	2.322	0.09
Very low (right), Very low (left)	1 vs 2*	− 3.252	0.011*
	1 vs 3	− 0.021	1
	2 vs 3*	3.23	0.012*

*< 0.05, **< 0.01.

### Comparison of performance between the double-target and single-target conditions

**Figure 5:**
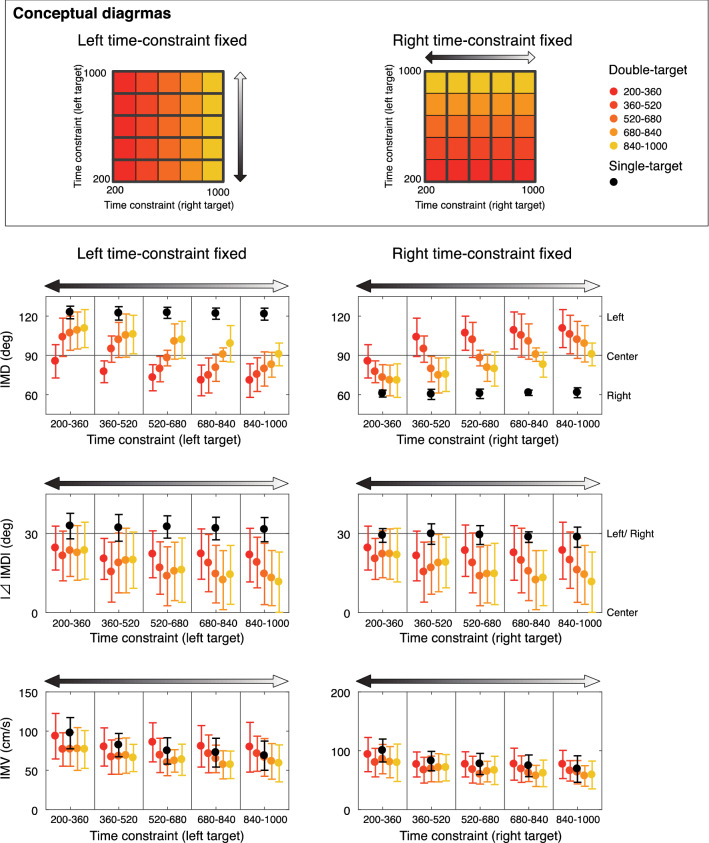
Comparison of the initial movement behavior among conditions. The upper, middle, and lower panels show inter-condition comparisons of the IMD, IMV, and |⊿IMD|, respectively. The circles and error bars show the means and standard deviations, respectively. The black-colored circles show the data for the single-target condition, and gradient circles from red to yellow show the data for the double-target condition. The horizontal axis of the left (/right) panel is classified by the level of time constraint of the left (/right) target, and the modulation according to the time constraint of the right (/left) target at each time constraint level is shown as a gradient. The color changes from red to yellow depending on the severity of the time constraint of the other target (red is tight, yellow is loose).

Figure 5 shows an inter-condition comparison of the initial movement direction (IMD), |⊿IMD|, and initial movement velocity (IMV). |⊿IMD| was defined as the absolute value of the angle difference between the IMD and the vertical vector, and it evaluated whether the initial movement was directed in the target direction or the center direction (with only signed indicators, the values would be the same if both targets were headed in the same frequency, or if center direction were headed at all trials). Red-yellow gradation and black circles denote the data for the double- and single-target condition, respectively. The panels in the left (/right) column show the modulation according to the time constraint of the right (/left) target at the same level of time constraint of the left (/right) target (for details, see the conceptual diagrams in the Figs. 4 and 5 and the captions).

Similar to the results observed in Fig. 4, from the upper and lower row panels in Fig. 5, it was shown that the initial movement deviated from center direction to the direction with a shorter time constraint. A two-way repeated-measures ANOVA (5 [time constraints on left target] × 5 [time constraints on right target]) of the IMD revealed significant main effects of time constraints on the left target (*F*_[4,44]_ = 23.296, η^2^_p_ = 0.679, *p* < 0.001) and time constraints on the right target (*F*_[4,44]_ = 7.697, η^2^_p_ = 0.412, *p* < 0.001) and a significant interaction (*F*_[16,176]_ = 3.669, η^2^_p_ = 0.25, *p* < 0.001). Post-hoc tests showed simple main effects of both left and right time constraints in all time constraint levels (right time constraints in 200–360 ms: *p* = 0.002 others: *p*s < 0.001).

Similarly, a two-way repeated-measures ANOVA (5 [time constraints on left target] × 5 [time constraints on right target]) of the |⊿IMD| revealed significant main effects of time constraints on the left target (*F*_[4,44]_ = 8.114, η^2^_p_ = 0.425, *p* < 0.001) and time constraints on the right target (*F*_[4,44]_ = 10.154, η^2^_p_ = 0.48, *p* < 0.001) and a significant interaction (*F*_[16,176]_ = 2.437, η^2^_p_ = 0.181, *p* = 0.002). Post-hoc tests showed simple main effects of left time constraints in 520–680 ms (*p* < 0.001), 680–840 ms (*p* < 0.001), and 840–1000 ms (*p* = 0.001), and those of right time constraints in 360–520 ms (*p* = 0.014), 520–680 ms (*p* < 0.001), 680–840 ms (*p* < 0.001), and 840–1000 ms (*p* < 0.001). From the modulation of IMD and |⊿IMD|, it was shown that the initial direction varies according to the combination of time constraints, similar to the results of the cluster analysis (Fig. 4).

Additionally, for both IMD and |⊿IMD|, it was shown that the initial movements in the double-target condition deviated in the central direction compared to those in the single-target condition at any time constraint level(upper and middle rows in Fig. 5). This was also indicated by the fact that the frequency of movement in the central direction was not zero at any time constraint level in the cluster analysis (Fig. 4). This meant that, within the current range, the participants did not completely ignore one of the targets, no matter how tight the time constraint was.

From the upper row panels in Fig. 5, it was found that the IMV was slower as the time constraint was longer. A two-way repeated-measures ANOVA (5 [time constraints on left target] × 5 [time constraints on right target]) of the IMV revealed significant main effects of time constraints on the left target (*F*_[4,44]_ = 22.456, η^2^_p_ = 0.671, *p* < 0.001) and time constraints on the right target (*F*_[4,44]_ = 15.436, η^2^_p_ = 0.584, *p* < 0.001) and no significant interaction (*F*_[16,176]_ = 1.363, η^2^_p_ = 0.11, *p* = 0.165). These results suggested that the initial velocity varies according to the time constraints and that the faster initial movement was executed when the time constraint was shorter.

**Figure 6:**
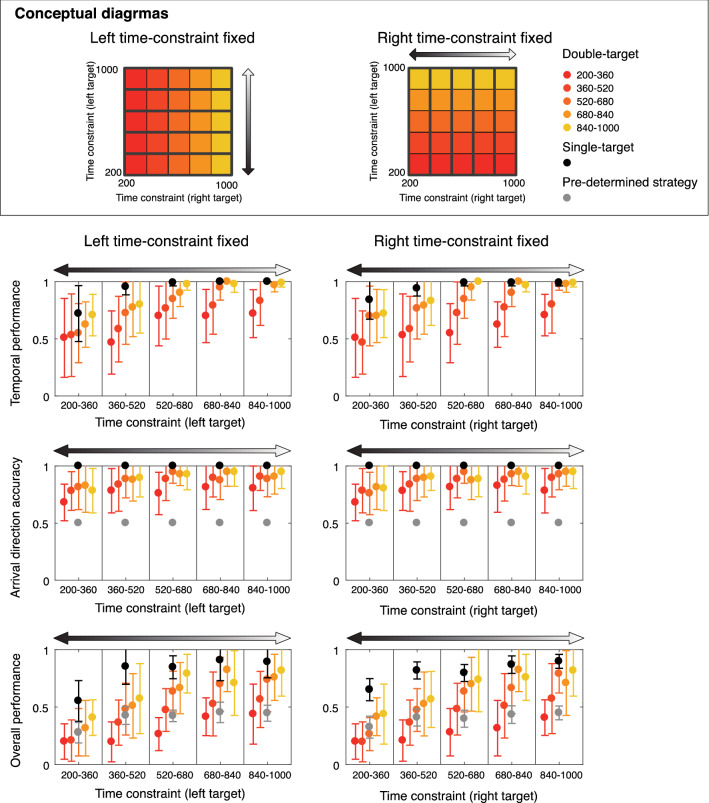
Comparison of performance (temporal accuracy, arrival target accuracy, overall performance) among conditions. The upper, middle, and lower panels show inter-condition comparisons of the temporal accuracy, arrival target accuracy, and overall performance, respectively. The circles and error bars show the mean and standard deviation, respectively. The black-colored circles show the data for the single-target condition. The gray-colored circles show the estimated performance of predeterminant strategy based on the data in the single-target condition. The gradient circles from red to yellow show the data for the double-target condition. The horizontal axis of the left (/right) panel is classified by the level of time constraint of the left (/right) target, and the modulation according to the time constraint of the right (/left) target at each time constraint level is shown as a gradient. The color changes from red to yellow, depending on the severity of the time constraint of the other target (red is tight, yellow is loose). In tight time-constraint condition (the leftmost time constraint level in each panel), the temporal accuracy in the double-target condition was significantly less than that in the single-target condition, and the arrival target accuracy in the double-target condition was significantly higher than the performance of predeterminant strategy estimated from the data of the single-target condition (gray-colored circles and lines). Additionally, in tight time-constraint condition (the leftmost time constraint level in each panel), the overall performance in the doubl-target condition was significantly less than the performance of the predeterminant strategy (gray-colored circles and lines). This deficit in performance may be due to excessive preference for the choice reaction even in the tight time-constraint conditions.

Figure 6 shows an inter-condition comparison of the temporal performance, arrival direction accuracy, and overall performance. The left (/right) column shows the performance modulation depending on the time constraint for the right (/left) target when the time constraint for the left (/right) target was the same level. If the same action was taken in the double-target condition as in the single-target condition, the chance level to reach the target was 50%. Therefore, for the arrival direction accuracy and overall performance, the gray color indicates the data of the single-target condition multiplied by 1/2 to estimate the performance when the predetermined strategy is adopted. The temporal performance in the double-target condition was confirmed to be lower than that in the single-target condition. Moreover, the arrival direction accuracy in the double-target condition was relatively higher than that in the single-target condition (50% accuracy), although it was slightly lower in the condition with severe time constraints. This indicated that the choice-reaction strategy was used more frequently than the predetermination strategy, and that even though the initial movement was directed in either target direction, the movement was eventually carried out in the direction of the final target.

Because the performance of the double-target condition seemed to be lower than that of the single-target condition under the tightest time constraint, an exploratory analysis was conducted to examine the effect of the other time constraint condition and the number of targets under the condition where one of the time constraints was the most severe. A two-way repeated-measures ANOVA (2[number of targets] × 5[time constraints]) on the overall performance revealed significant main effects of both the number of targets (left fixed: *F*_[1,11]_  = 28.132, η^2^_p_ = 0.719, *p* < 0.001, right fixed: *F*_[1,11]_ = 16.628, η^2^_p_ = 0.602, *p* = 0.002) and time constraints (left fixed: *F*_[4, 44]_ = 5.877, η^2^_p_ = 0.348, *p* < 0.001; right fixed: *F*_[4,44]_ = 6.019, η^2^_p_ = 0.354, *p* < 0.001). These results suggest that the overall performance in the double-target condition seemed to be higher than that of the predetermined strategy when the time constraint was relatively loose, but the predetermined strategy performed better when the time constraint was severe (especially when the red-colored data were checked).

A two-way repeated-measures ANOVA (2[number of targets] × 5[time constraints]) on the temporal accuracy revealed significant main effects of both the number of targets (left fixed: *F*_[1,11]_ = 33.968, η^2^_p_ = 0.755, *p* < 0.001, right fixed: *F*_[1,11]_ = 29.335, η^2^_p_ = 0.727, *p* < 0.001) and time constraints (left fixed: *F*_[4,44]_ = 7.746, η^2^_p_ = 0.396, *p* < 0.001; right fixed: *F*_[4,44]_ = 6.019, η^2^_p_ = 0.413, *p* < 0.001). There was a significant interaction only in right fixed (*F*_[4,44]_ = 3.038, η^2^_p_ = 0.216, *p* = 0.027). Post-hoc tests revealed that there were significant simple main effects of number of targets in four time constraint levels (*p*s < 0.01, in 360–520 ms, 520–680 ms, 680–840 ms, and 840–1000 ms). These results suggest that the temporal accuracy in the double-target condition was less than that in the predetermined strategy.

The arrival direction accuracy was compared to the accuracy in the single-target condition multiplied by 1/2 for comparison with the predetermined strategy (which was 0.5 for the previous participant, since all participants did not make a directional error in the single-target condition). A two-way repeated-measures ANOVA (2[number of targets] × 5[time constraints]) on the arrival direction accuracy revealed significant main effects of the number of targets (left fixed: *F*_[1,11]_ = 59.091, η^2^_p_ = 0.843, *p* < 0.001, right fixed: *F*_[1,11]_ = 48.172, η^2^_p_ = 48.172, *p* < 0.001) and no significant main effect of time constraints (left fixed: *F*_[4,44]_ = 1.365, η^2^_p_ = 0.11, *p* = 0.261; right fixed: *F*_[4,44]_ = 1.781, η^2^_p_ = 0.139, *p* = 0.15). These results suggest that the arrival direction accuracy in the double-target condition was higher than that in the predetermined strategy.

Taken together, comparisons of the performance between the double-target condition and the single-target condition (predetermined strategy) revealed that the emphasis on spatial accuracy slowed down the arrival time, resulting in poor temporal performance. Moreover, the low temporal performance in double-target condition had such an impact that it decreased the overall performance to below that of the single-target condition. These results indicated that even in situations where time constraints were tight and a predetermined strategy was desirable, a selective strategy was adopted.

## Discussion

The current study examined how an initial movement is selected for a given time constraint for each potential target and whether the selection of the strategy for a given time constraint is desirable in terms of success probability. The results showed that the initial movement evaluated by the motor variables 100 ms after the movement onset varied according to the given time constraint, suggesting that participants adjust their behavior depending on the combination of time constraints. Specifically, in situations where the severities of the time constraints were comparable, the initial movement was directed toward the average direction of the potential targets, and in situations where there was a substantial difference in the severities of the time constraints, the initial movement was directed toward the target direction with a more severe time constraint. In addition, a comparison of the initial movement and performance between the double-target and single-target conditions revealed that a selective strategy was adopted even when a predetermined strategy was desirable.

Previous studies have reported that in the presence of multiple potential targets, humans vary the initial movement depending on the condition of motor planning. For instance, although the participants selected an initial movement in an intermediate direction at a relatively slow movement speed, such an initial movement was no longer selected, and the initial movement headed linearly to one of the potential targets at the fast movement speed required ^8^. These results indicate that the planning of the initial movement under multiple potential targets reflects the optimization of the task. A recent study reported that motor planning (in particular, preparation of feedback responses) is adjusted in a utility-dependent manner when asymmetry exists in the utility assigned to the target^30^. These studies showed that motor planning may purposefully depend on restrictions of motor execution (e.g., speed and direction) and target information (e.g., value and size).

As an extension of the above study, the current study experimentally manipulated the time constraints assigned to each potential target and investigated the modulation of the initial movement according to the combinations of time constraints. The results showed that the velocity and direction of the initial movement were modulated according to the combination of time constraints. Specifically, the participants generally initiated their movements in the middle direction of the potential target in conditions with equal time constraints. In the conditions where the time constraints differed, participants generally initiated their movements in the direction of the target with the shorter time constraint and corrected their movements when the target with the longer time constraint was correct. This strategy of initially prioritizing the target with a tighter time constraint and correcting the movement if the other target was the true target is considered to be a behavior that reflects the task demands: targets with short time constraints need to be reached quickly, while more time can be taken for targets with longer time constraints.

One possible explanation for the modulation of initiation behavior to multiple potential targets is task optimization or reward maximization^7,8^. However, the extent of optimality in such tasks has not yet been tested. In particular, an important aspect of strategy selection in a time-urgent situation is the decision to use a predetermined strategy or a choice-reaction strategy. In this case, optimality lies not in the ability to choose the more desirable strategy under certain extreme conditions (e.g., conditions where time constraints are too tight or too loose), but in the ability to switch strategies appropriately in situations where the expected outcomes are equivalent. The present study exploratively investigated whether switching between the predetermined and the choice-reaction strategy was appropriate by comparing the initiating behavior and performance between the single-target and double-target conditions. The results revealed that the temporal performance in the double-target condition was lower than that in the predetermined strategy (i.e., estimated by the behaviors in the single-target condition), and the probability of reaching the correct target direction was higher than that in the predetermined strategy, resulting in poorer outcomes when the time constraint was severe. Additionally, the direction of the initiating action was not always directed toward one target, although the probability of being directed toward the central direction decreased in the condition with severe time constraints. These results suggest that even in conditions where a predetermined strategy is desirable, there is a bias toward the choice-reaction strategy that results in poor performance.

We propose several possible causes for this preference bias in the choice-reaction strategy. First, inaccurate estimation of own sensorimotor system may lead to limited optimality. The normative perspective in the current study assumes that the decision-maker is aware of the exact relationship between the strategy and the outcome of the movement. However, it is unclear whether the participants actually recognized this relationship completely and accurately. Indeed, in a timing-coincidence task, the recognized outcome of one's own action was reported to be perceived closer to the success direction than the actual one^31^. Moreover, motor variance also tended to be more under-recognized than it actually is^32,33^, and there is consistency among individuals in the pattern of deviations from the optimal solution. This may be due to the biased recognition of one's own sensorimotor system.

Second, it is conceivable that participants found more value in spatial accuracy than in temporal accuracy. In the current task, the final outcome (obtaining a score) was determined by two dimensions: space and time. In tasks involving multidimensional elements in the final outcome, interpretation of the outcome of each dimension in an integrated manner remains a challenge. If participants try to reach both potential targets, they can reach the direction of the correct target even if they fail to reach it in time. In contrast, with a predetermined strategy, the participants will go in a different direction from the correct target in approximately half of the trials. With the choice-reaction strategy, even if they could not reach the target in time, they may find value in the fact that they were able to eventually reach the target location. Correspondingly, even if they had made it in time, they may have found a loss in reaching a direction where the target did not exist. Future research is necessary to understand how each factor is recognized and integrated when both time and space affect success or failure, as in the current task.

Another possibility is that the subjective utility of the strategy led to a systematic deviation from the better strategy. In a predetermined strategy, the outcome of the movement depends on external factors. As we underestimate the value of a "lucky hit," we may underestimate the value of a strategy that relies on such randomness. This may reflect an unwillingness to devote effort to action if there is a high likelihood that it will be futile or reflect a low estimate of the value of the action if it succeeds or a high estimate of the loss if it fails. If such a cognitive bias is at work, it could be a strong impediment to strategy optimization in situations with insufficient time to execute the targeted action. Importantly, the same tendency has been consistently observed in our previous studies^29^ and in other recent studies^28,34^, necessitating the identification of its causes.

Finally, in the current optimization norms, it is assumed that all possible motor plans are simulated, but all motor plans may not necessarily be simulated in a finite amount of time. One obvious possibility is that the participants' motor plans reflect an optimization of the entire task, rather than the optimization of only a given condition. In fact, in the range of conditions in the present experiment, the more desirable condition was the one in which the participants were trying to reach the two targets. Therefore, a choice-reaction strategy may be adopted even in a minority of conditions where a predeterminant strategy is desirable. It is also known that humans tend to overestimate the loss caused by choosing an option that is not currently implemented and prefer to maintain the status quo^35^. Our daily strategy selections are made in the face of constantly changing circumstances. In such situations, we may not be optimizing locally for a given condition, but rather globally, including a wider range of time scales. Future studies are required to clarify the type of optimization performed when successively dealing with various situations.

## Conclusion

The current study investigated how an initial movement is selected for a given time constraint for each potential target and whether the selection of the strategy for a given time constraint is desirable in terms of success probability. The results showed that the motor patterns were modulated according to the combination of given time constraints. However, under tight time constraints, the success rate in the double-target condition was less than the single-target condition, indicating that the initial movement for multiple potential targets with independent time constraints can be modified, but the planning is suboptimal. It was considered that this could be due to the asymmetry between spatial and temporal success in value representations, as well as inaccuracies in the estimation of their own abilities. Future research should shed light on the factors that prevent people from optimizing their movement strategies. At the same time, it is necessary to develop learning and intervention methods to promote the optimization of movement strategies.

## Supplementary Information

Supplementary Information.
