# Supplementary material for: Modulation of initial movement for double potential targets with specific time constraints
Source: Sci Rep. 2021 Nov 18;11:22492. doi: 10.1038/s41598-021-01777-3 (PMC8602633; doi:10.1038/s41598-021-01777-3)

Supplementary Information

Modulation of initial movement for double potential targets with specific time constraints

Ryoji Onagawa [1,2,3] and Kazutoshi Kudo[1]

1. Laboratory of Sports Sciences, Department of Life Sciences, Graduate School of Arts and Sciences, The University of Tokyo, Tokyo, Japan.
2. Research Fellow of Japan Society for the Promotion of Science, Tokyo, Japan.
3. Faculty of Science and Engineering, Waseda University, Tokyo, Japan

Supplementary Figures

The following figures show the trajectories of each participant depending on the combination of time constraints. For each participant, trajectories are drawn according to the 3×3 combination for time constraints, and the time constraints are indicated by indicators above the targets (Same classification as Fig. 2 in the main text).

ID = A

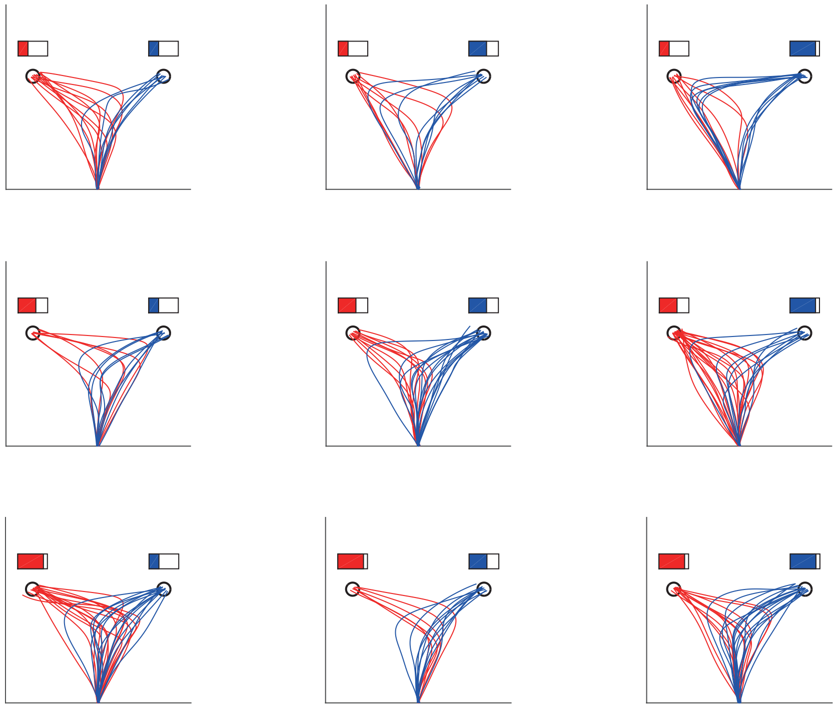

ID = B

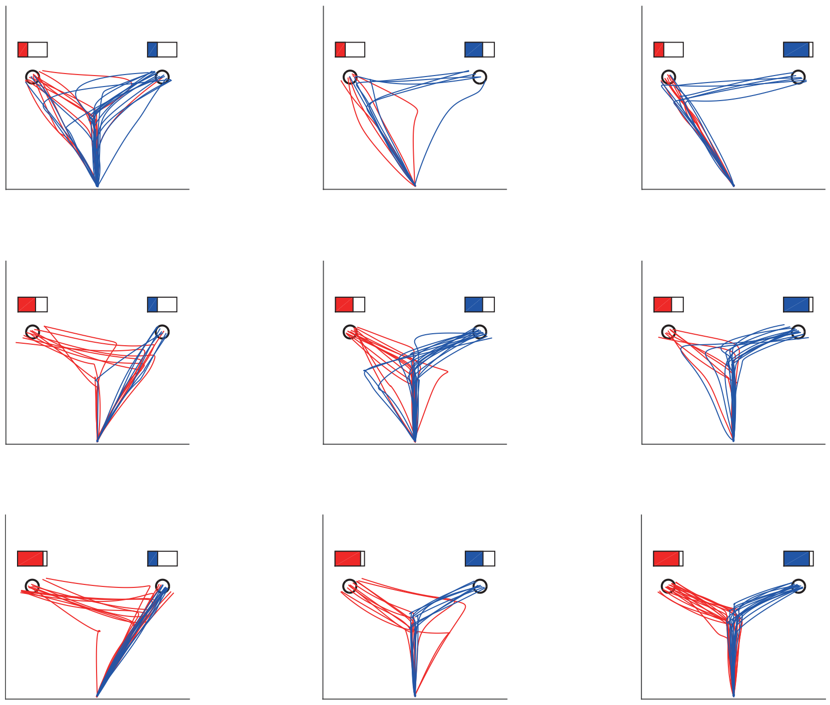

ID = C

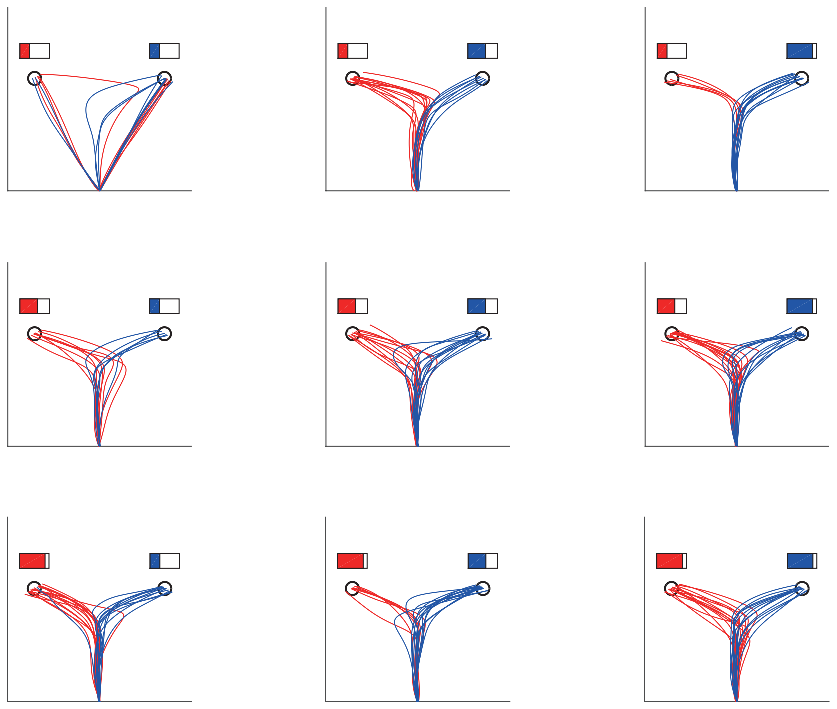

ID = D

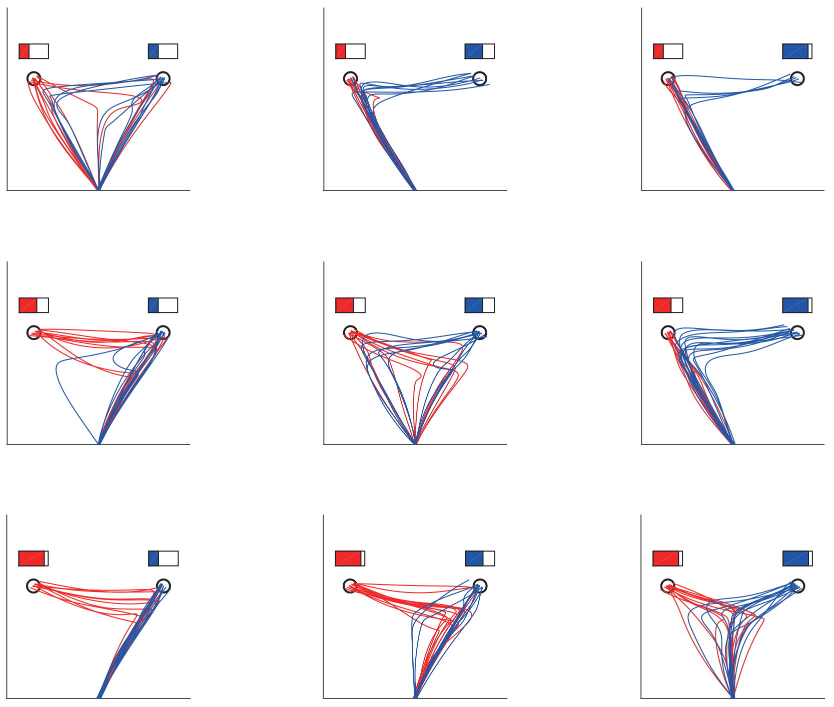

ID = E

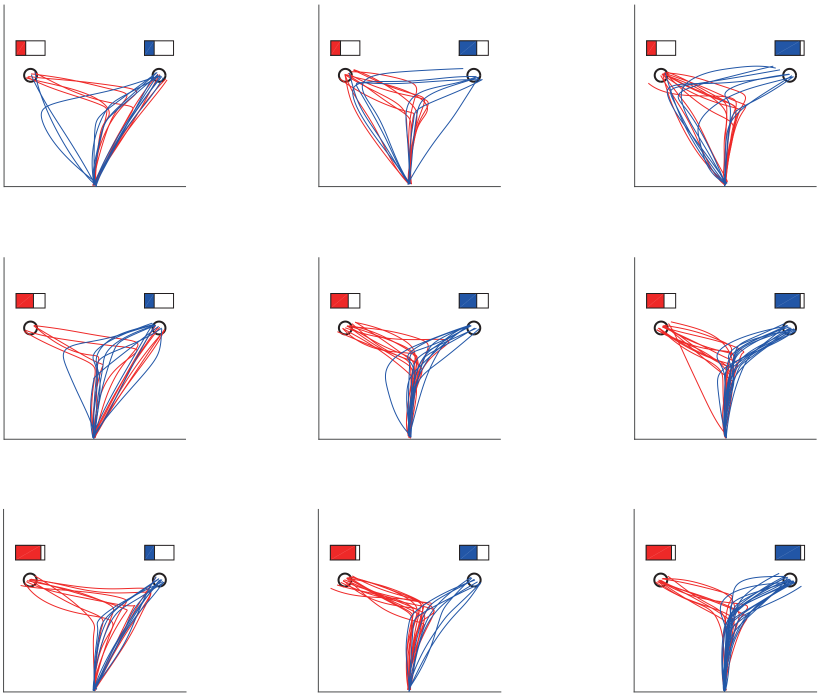

ID = F

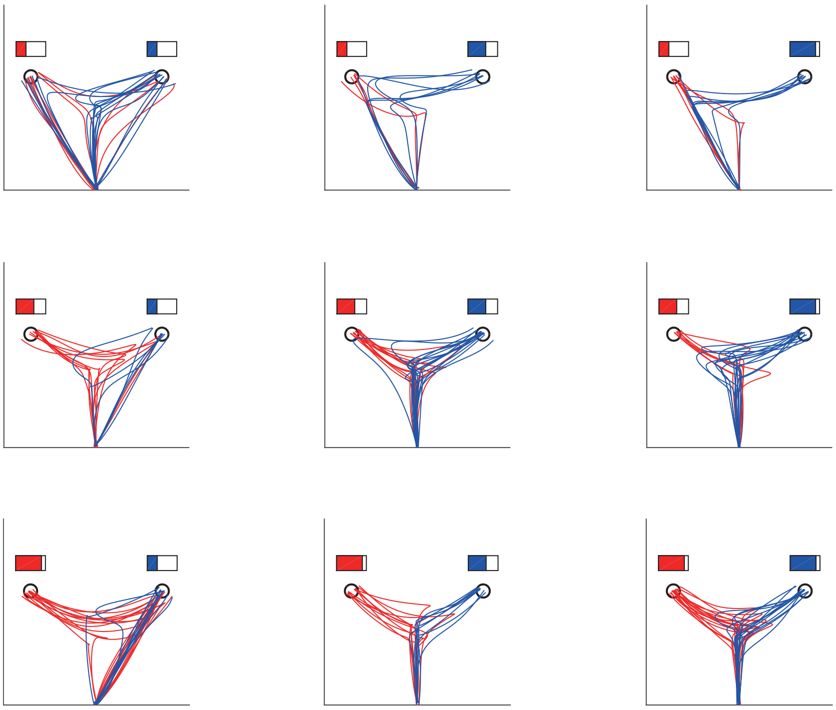

ID = G

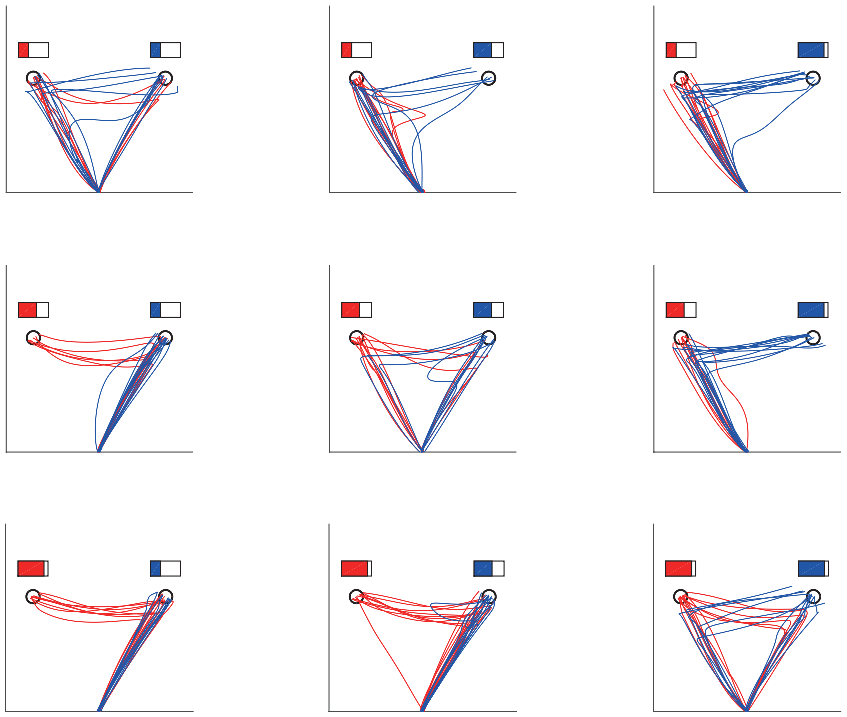

ID = H

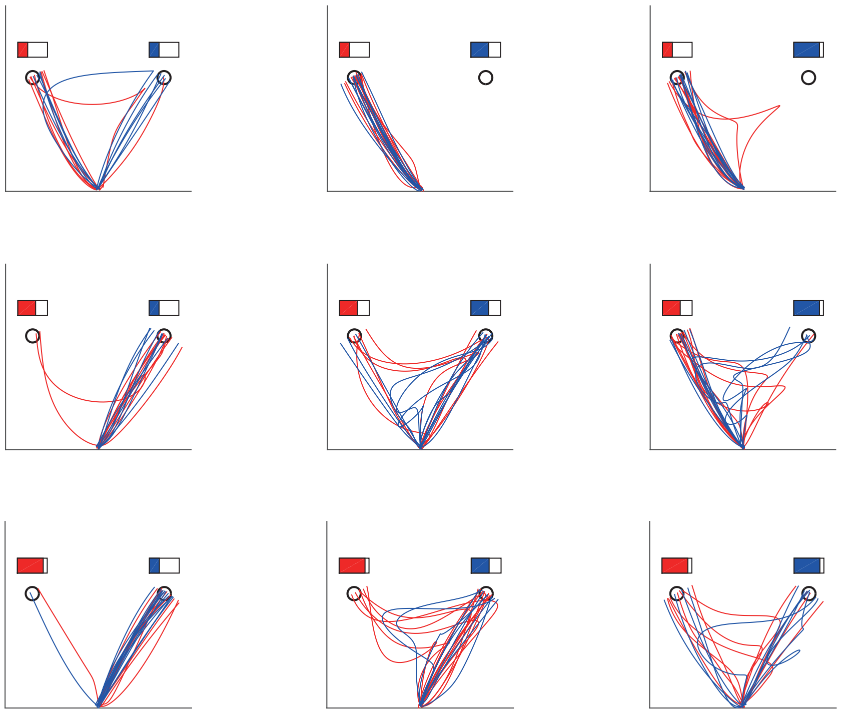

ID = I

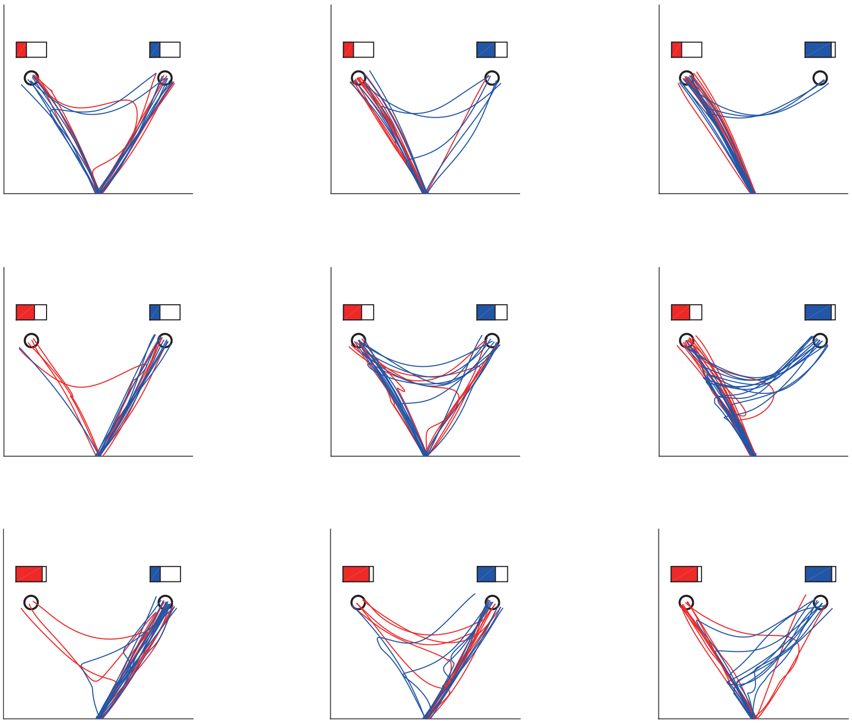

ID = J

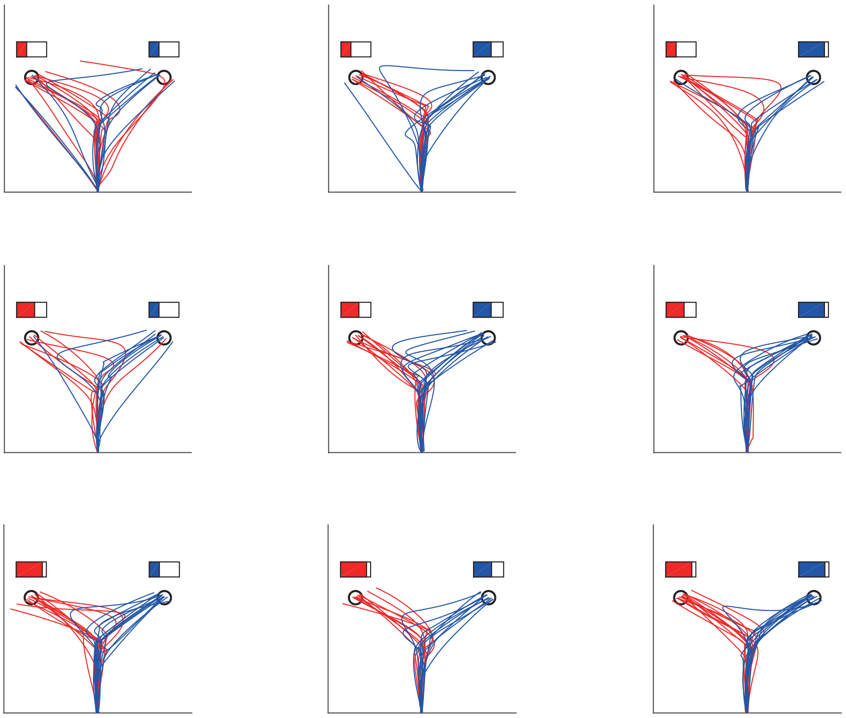

ID = K

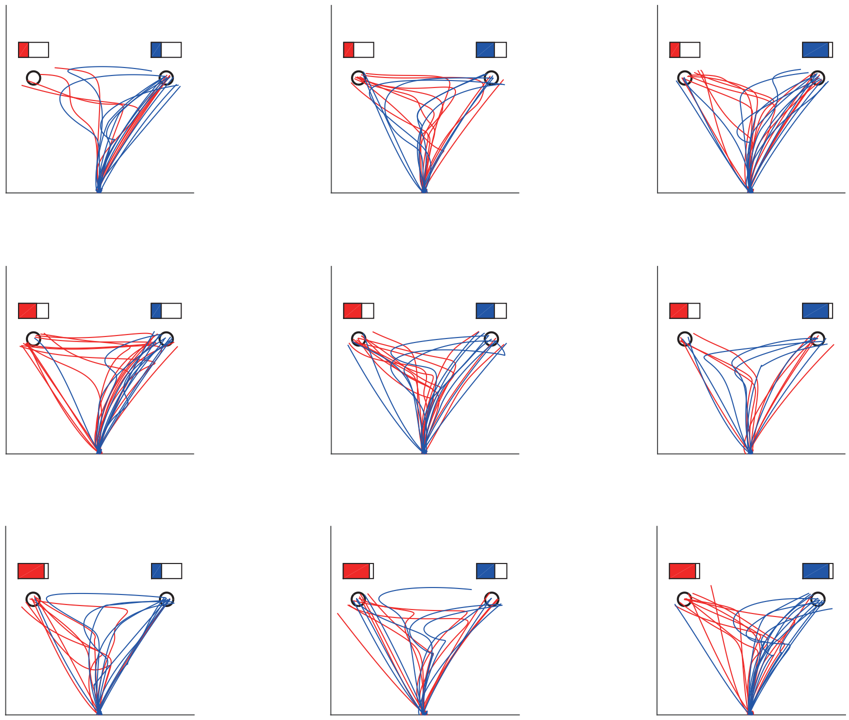

ID = L

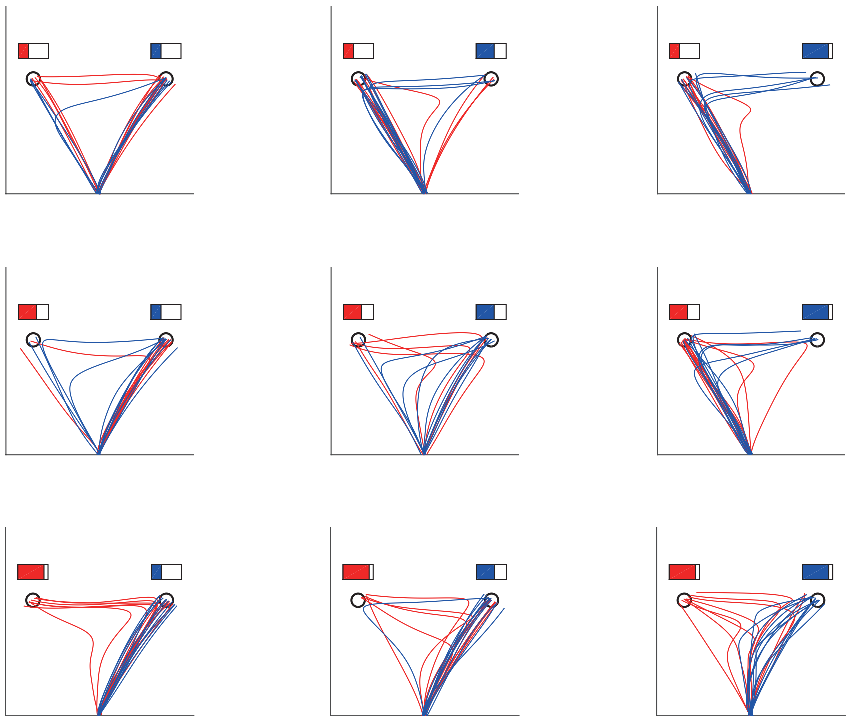

Supplement: Supplementary file 1 — Supplementary Information. [file 41598_2021_1777_MOESM1_ESM.pdf]
